# Prevalence of onchocerciasis after seven years of continuous community-directed treatment with ivermectin in the Ntui health district, Centre region, Cameroon

**DOI:** 10.11604/pamj.2020.36.180.20765

**Published:** 2020-07-14

**Authors:** Estelle Makou Tsapi, Françoise Guemgne Todjom, Guy-Armand Gamago, Josué Wabo Pone, Félicité Flore Djuikwo Teukeng

**Affiliations:** 1 Evangelical University of Cameroon, Bandjoun, Cameroon,; 2 Research Unit of Biology and Applied Ecology, Faculty of Science, University of Dschang, Dschang, Cameroon,; 3 Faculté des Sciences de la Santé, Université des Montagnes, Bangangté, Cameroon

**Keywords:** Cameroon, epidemiology, hypoendemicity, onchocerciasis, prevalence

## Abstract

**Introduction:**

onchocerciasis is one of the major infectious diseases caused by Onchocerca volvulus. This parasite is responsible for chronic cutaneous and ocular diseases affecting more than 37 million people of whom 99% are in Africa. The study was conducted in the health district of Ntui from June to September 2016 to determine the prevalence of O. volvulus infection after seven years of massive administration of ivermectin.

**Methods:**

two cutaneous snips were made at the iliac crests level in volunteers. These tissues were incubated in physiological saline water and were examined for parasitological investigations in the laboratory.

**Results:**

a total of 310 participants were randomly selected, of whom 170 (54.8%) were women and 140 (45.1%) were men aged 6 to 83 years, thus giving a sex ratio of 1.2 in favour of women. After parasitological analysis, 26 participants had microfilaraemia, of whom 15 (10.7%) were men and 11 (6.4%) were women. The most infected age group was 16 to 26 years (12.5%). The highest infection rates were found among farmers (11%) and participants living in the village of Essougly (26.6%). No significant differences in prevalence values between the different groups were noted, whatever the parameter considered.

**Conclusion:**

the prevalence of onchocerciasis in the health district of Ntui has declined from a hyperendemic to a hypoendemic state after seven years of massive administration of ivermectin. However, careful monitoring of onchocerciasis should be continued to prevent the area from returning to its original hyperendemicity.

## Introduction

Onchocerciasis is a neglected tropical disease of the skin and eyes caused by the filarial nematode *Onchocerca volvulus*, which lives in the lymphatic and subcutaneous spaces of humans. It is transmitted by the bites of infected black flies belonging to the genus *Simulium*. In the world, it is the second infection that causes blindness. Onchocerciasis is also responsible for skin disease such as unsightly skin lesions, debilitating itching and excess mortality among highly infected people. It is also a major risk factor for epilepsy and nodding syndrome [1,2]. Because of its negative impact on health, this public health problem was important in tropical Africa, Latin America and Yemen with more than 40 million people infected before the launch of large-scale control and an at-risk population of more than 160 million of which more than 99% live in Africa [3,4]. In 1987, the use of a safe anthelminthic: ivermectin (Mectizan™) has radically changed the fight against onchocerciasis. Ivermectin administration at regular intervals has, in the long term, interrupted transmission of new infections with *O. volvulus* in endemic foci [5,6].

Mass drug administration (MDA) campaigns repeatedly conducted through control programs have eliminated the disease in four Latin American countries between 2013 and 2016, namely Colombia, Ecuador, Guatemala and Mexico [7,8]. In 1995, at the launch of the African Program for Onchocerciasis Control (APOC), 37 million people were assumed to be infected. This program was launched with the main objective of controlling onchocerciasis as a public health problem in those African countries which were not covered by the Organization for the Prevention of Blindness (“Organisation pour la Prévention de la Cécité”) and supported more than 80% of the global burden of onchocercal disease [4]. This led to a significant improvement in the coverage of MDA treatment. After 30 years of MDA with Mectizan™, onchocerciasis transmission was interrupted in some African foci by massive administration of ivermectin once or twice a year. The feasibility of eliminating onchocerciasis through long-term mass treatment with ivermectin in Africa has been demonstrated in Mali and Senegal [5,9]. In Cameroon, a significant reduction in the prevalence of infection in children and adults after 5 to 13 years of annual community treatment with ivermectin has been demonstrated [10].

However, onchocerciasis remains a major concern in several endemic zones [11], with prevalence values above 60% in the centre, littoral and west in 2011 [12]. Indeed, 62% of rural people living in these endemic areas are threatened, i.e. about 9 million Cameroonians of whom 5 million are infected by worms and 30,000 blind [13]. In 2009, the prevalence of onchocerciasis in the forest-savanna transition zone in the Ntui health district was 26.3% [14]. Although there is some evidence to suggest that the disease is about to be eradicated in several districts of Cameroon [15], extensive epidemiological and entomological investigations are still needed to assess the disease situation in order to detect infection in human population and vector insect samples. The objective of the present survey was therefore: to determine the prevalence of *O. volvulus* in the Ntui health district after seven years of Continuous Community-Directed Treatment Intervention (CDTI) with ivermectin and; to assess whether treatment with ivermectin should be continued at the same or different periodicity depending on the prevalence in people and black flies.

## Methods

**Study area:** this study was designed as a cross-sectional prevalence study and was conducted from June to September 2016 among residents of Ntui, a Cameroonian town located in the Centre region. Ntui is also the main town of the Mbam and Kim division. This city is located in the equatorial zone (42°27'00" N, 11°38'00" E). The relief is marked by the presence of plains, hills, valleys and slopes. The climate is subequatorial with a tropical tendency: it is characterized by a long dry season from mid-November to the end of March, a short rainy season from mid-March to mid-May, a short dry season from mid-June to mid-July and a long rainy season from mid-August to mid-November [16]. The average annual temperature in this zone is 26°C, with a maximum reaching 37°C in January [16]. The hydrographic network includes the Sanaga river and four tributaries: Meloko, Mpiem, Obagne and Ossombo. The main activity of population is agriculture. A previous survey in this area showed a high prevalence of onchocerciasis: 26.3% in 2009 [14]. Since that date, no investigation has been conducted despite an annual mass drug administration with ivermectin in that locality.

**Selection of participants:** this study was carried out at the Ntui District Hospital. Eleven health areas and 30 functional sanitary facilities (18 public and 12 private) are present in this district [17]. The minimum sample size was estimated using the report by Naing *et al*. [18] according to the formula.

Where N, minimum sample size; Z, standard value; P, expected prevalence of onchocerciasis in the study area; d, precision of error at 5% with a 95% confidence interval. As Z=1.96, P=26.3% (the prevalence in the area in 2009) and d=5%, N=298. Each participant received a pre-tested questionnaire and was also interviewed about his socio-demographic, clinical and parasitological data. His (or her) name, age, sex, home and occupation were recorded. Information was also provided on the symptoms of onchocerciasis. During the interview, the children were asked in their mother language (“mbulu”) to ensure the reliability of the information.

**Protocol of investigations:** a total of 310 people were enrolled in the study. In each village, the first task was to sensitize the whole population. The symptoms noted during the interrogation were pruritus (skin and eyes), filarial scabies, depigmentation of the skin, lacrimation, blurred vision, decreased visual field and/or gene in the light. After the population census, two biopsies were performed on each participant, one at the left iliac crest and the other at the right iliac crest. Each area to be biopsied was disinfected with 70% alcohol and the skin was lifted (approximately 1 to 2mm) with a needle to collect a small piece of skin with a razor blade. Each skin fragment was then placed in a coded tube containing physiological saline water and left at outdoor temperature. Samples collected from a household were sent to the hospital laboratory prior to visit the next household. The fresh preparation was left to stand and was analyzed after 30 min and after 24h under a light microscope at low magnification (10x and 40x) to detect microfilariae. Individuals with biopsies containing microfilariae after 30 min were considered infected with *O. volvulus*.

**Parameters studied and statistical analysis:** the overall prevalence of *O. volvulus* infection was expressed in relation to the age of participants (from 6 to more than 46 years), their sex, their occupation and the village in which they lived along the Sanaga river. The collected data were entered into Microsoft Excel 2013 and imported into the SPSS 20.0 software. Prevalence values were compared using the chi-squared test. The differences were considered significant at p<0.05.

**Ethical considerations:** the protocol in this study was approved by the Institutional Ethics Committee of the Evangelical University Institute of Cameroon (IUEC).

## Results

A total of 310 participants were randomly selected. Of the 310 samples collected, 26 (8.3%) were infected with *O. volvulus*. The prevalence of infection was 10.7% (15/140) in boys and men, while it was 6.4% (11/170) in girls and women. The 16-26 year group (Table 1) was the most infected (12.5%). A gradual decrease in the prevalence was noted in the other age groups: 26-36 yrs (11.2%), > 46 yrs (8%), 36-46 yrs (7.6%) and 6-16 yrs (2.2%). In the population studied (Table 2), the infection was high among farmers (10.7%), followed by housewives (8.6%) and schoolchildren (2.2%). No significant difference between the prevalence values was noted, whatever the factor studied. The prevalence of infection in relation to the housing of participants is given in Table 3. Participants from the Essougly village were the most infected (26.6%), followed by those from Kela (23.5%), Ndowe (15.7%), Nachtigal (13.3%) and Nkolve (11.7%). In each of the following villages: Ehondo, Kake, Mbandona, Mbanga, Nkouloutou and Onguesse, the prevalence of infection was less than 10%. No infected patient was noted in the other three villages. No significant difference between the infection rates recorded in the 11 villages was noted.

**Table 1:** prevalence of *Onchocerca volvulus* infection in the 310 samples in relation to the age of participants

Age groups	Number of cases examined	Number of positive cases	Prevalence (%)
[6-16]	44	1	2.2
[16-26]	40	5	12.5
[26-36]	62	7	11.2
[36-46]	39	3	7.6
[> 46]	125	10	8.0
Total	310	26	8.3

**Table 2:** prevalence of *Onchocerca volvulus* infection in relation to the occupation of participants

Profession	Number of cases examined	Number of positive cases	Prevalence (%)
Farmers	196	21	10.7
Housewifes	46	4	8.6
Schoolchildren	44	1	2.2
Officials	7	0	0
Masons	6	0	0
Drivers	2	0	0

**Table 3:** prevalence of *Onchocerca volvulus* infection in relation to the village where the participants lived

Village	Distance between each village and the river (km)	Number of cases examined	Number of positive cases	Prevalence (%)
Essougly	1	15	4	26.6
Ndowe		38	6	15.7
Nachtigal		15	2	13.3
Nkolve		17	2	11.7
Mbanga		22	2	9.0
Onguesse		23	1	4.3
Nkouloutou	2	21	2	9.5
Kake		14	1	7.1
Mbandonna		18	1	5.5
Kéla	3	17	4	23.5
Ehondo		35	1	2,8
Ndjame	11	14	0	0
Betamba	12	22	0	0
Ntui	20	21	0	0

## Discussion

The objective of the present survey was to assess the prevalence of *O. volvulus* in the district of Ntui after seven years of Continuous Community-Directed Treatment Intervention (CDTI) with ivermectin. Our results show an overall prevalence of 8.3%. The prevalence of onchocerciasis in the study area is low, despite the presence of several fast-flowing rivers, black flies and an atmosphere favouring the interaction between farmers and biting flies. This result may be attributed to distribution of ivermectin for even years to eradicate onchocerciasis in this study area. Indeed, annual or semi-annual ivermectin treatments of populations in onchocerciasis endemic areas have proved to be an effective strategy for controlling the disease [19]. This low prevalence is similar to that (7.5%) reported by Katabarwa *et al*.in Uganda [20], but is lower than those reported by Same Ekobo *et al*.(13.2%) during the construction of the Lom Pangar dam in East Cameroon [21], by Kueté *et al*.(26.3%) in the central region of Cameroon [14] or by Osue *et al*.(37.9%) in Nigeria [22]. The difference observed in these studies can be due to the fact that populations have more or less taken the annual Mectizan™ treatment.

Compared to women, men were more infected in the present study (10.7% versus 6.4%). Similar results were reported by Wogu and Okaka [23] in the town of Okpujeau in Nigeria (27.5% for men and 20% for women) and by Kamga *et al*. [24] in the Fundong health district in north-west Cameroon (2% for men and 1.5% for women). The fact that men are more infected than women can be justified by their daily activities. Although women and men are engaged in the same field works, men during their activity are less likely to cover their body, which exposes them more to black fly bites since these flies have diurnal activities [25]. In the present study, the most infected age group with *O. volvulus* was 16 to 26 years with a prevalence of 12.5%, whereas the least infected (2.2%) was aged 6 to 16 years. These results are different from those reported by Uttah (2010) in the Imo river watershed in Nigeria [26]. According to this author, people aged 60 years and more were the most infected (70.4%), while the least infected (13.9%) concerned patients aged 20 to 39 years [26]. The difference between these two studies could be explained by the fact that people of 16 to 26 years were the single persons which worked the most in the plantations of the Ntui locality.

Farmers (10.7%) were more infected, followed by housewives (8.6%) and schoolchildren (2.2%). These observations are similar to those found by Wandji *et al*. in the Konye, Mamfe, Eyumojok and Kumbadans districts of south-west Cameroon [27]. In fact, black flies did not enter the houses, but stung outside during the day along the river banks [28]. Most infected participants (26.6%) came from the Essougly village, followed by those of Kela, Ndowe, Nachtigal, Nkolve, Nkouloutou, Mbanga and Ehondo (23.5%, 15.7% 13.3%, 11.7%, 9.5%, 9% and 7.1%). All these villages are located less than 10km from the Nachtigal waterfalls and are hyperendemic for onchocerciasis because the distance between their village and this river was negatively correlated with the prevalence of *O. volvulus* infection and the black flies did not fly more than 6km [29]. This high prevalence could be explained by the fact that the population of the Essougly village was more present in the cocoa plantations, which are very close to the Sanaga river, as well as by its high vegetation.

## Conclusion

The overall prevalence of human infections by *O. volvulus* (8.3%) in the district of Ntui has considerably decreased over time. Similarly, the male sex, the age group 16-26 years and farmers were the most infected. The massive administration of ivermectin during seven successive years thus reduced the transmission of the disease. Consequently, the prevalence of *O. volvulus* infection in this health district has decreased from a hyperendemic to a hypoendemic state. However, careful monitoring of onchocerciasis should be continued to prevent the area from returning to its original hyperendemicity.

### What is known about this topic

- In Cameroon, a significant reduction in the prevalence of infection in children and adults after 5 to 13 years of annual community treatment with ivermectin has been demonstrated since 2003;
- Onchocerciasis remains a major concern in several endemic zones, with prevalence values above 60% in the centre, littoral and west in 2011.

### What this study adds

- The massive administration of ivermectin during seven successive years has reduced the transmission of the disease;
- The prevalence of onchocerciasis in the health district of Ntui has declined from a hyperendemic to a hypoendemic state.
